# A Practical Guide to Manual and Semi-Automated Neurosurgical Brain Lesion Segmentation

**DOI:** 10.3390/neurosci5030021

**Published:** 2024-08-02

**Authors:** Raunak Jain, Faith Lee, Nianhe Luo, Harpreet Hyare, Anand S. Pandit

**Affiliations:** 1 UCL Medical School, University College London, London WC1E 6DE, UK; raunak.jain.19@ucl.ac.uk (R.J.); faith.lee.18@ucl.ac.uk (F.L.); t.luo.17@ucl.ac.uk (N.L.); 2 Lysholm Department of Neuroradiology, National Hospital for Neurology and Neurosurgery, London WC1N 3BG, UK; h.hyare@ucl.ac.uk; 3 Victor Horsley Department of Neurosurgery, National Hospital for Neurology and Neurosurgery, London WC1N 3BG, UK; 4 High-Dimensional Neurology, Institute of Neurology, University College London, London WC1N 3BG, UK

**Keywords:** segmentation, education, radiomics, meningioma, glioblastoma

## Abstract

The purpose of the article is to provide a practical guide for manual and semi-automated image segmentation of common neurosurgical cranial lesions, namely meningioma, glioblastoma multiforme (GBM) and subarachnoid haemorrhage (SAH), for neurosurgical trainees and researchers. Materials and Methods: The medical images used were sourced from the Medical Image Computing and Computer Assisted Interventions Society (MICCAI) Multimodal Brain Tumour Segmentation Challenge (BRATS) image database and from the local Picture Archival and Communication System (PACS) record with consent. Image pre-processing was carried out using MRIcron software (v1.0.20190902). ITK-SNAP (v3.8.0) was used in this guideline due to its availability and powerful built-in segmentation tools, although others (Seg3D, Freesurfer and 3D Slicer) are available. Quality control was achieved by employing expert segmenters to review. Results: A pipeline was developed to demonstrate the pre-processing and manual and semi-automated segmentation of patient images for each cranial lesion, accompanied by image guidance and video recordings. Three sample segmentations were generated to illustrate potential challenges. Advice and solutions were provided within both text and video. Conclusions: Semi-automated segmentation methods enhance efficiency, increase reproducibility, and are suitable to be incorporated into future clinical practise. However, manual segmentation remains a highly effective technique in specific circumstances and provides initial training sets for the development of more advanced semi- and fully automated segmentation algorithms.

## 1. Introduction

Image segmentation algorithms are powerful tools for the delineation of regions of interest in medical images obtained through various modalities such as magnetic resonance imaging (MRI) and computed tomography (CT) [1]. Segmentation allows for the efficient delineation of pathology spread and border, to define the three-dimensional spatial characteristics of the lesion and the use of radiomics to determine clinical lesion characteristics such as volume, intensity and shape [2,3,4]. These features are valuable in clinical practise to allow for the determination of treatment planning, surgical approach, prognosis and, in the long-term, follow-up of patients with neurosurgical brain lesions [5,6,7].

Segmentation methods can be broadly divided into manual, semi-automated and fully automated types, depending on the level of involvement from the segmenter [8]. Manual segmentation describes the hand-crafted process of outlining structures in medical images in a slice-by-slice manner [9]. Semi-automated segmentation relies on pre-coded computer algorithms for initial segmentation and requires manual inspection and editing afterwards [10]. Fully automated segmentation typically employs machine learning for algorithm development and aims to minimise the manual input [11].

Medical image segmentation can be undertaken on various software including ITK-SNAP, Seg3D, Freesurfer and 3D Slicer [12]. While manual, semi- and fully automated techniques have been widely used in research for common neurosurgical conditions like brain tumours [13], subarachnoid haemorrhage [14] and hydrocephalus [15], their clinical and educational potential in neurosurgery remains undervalued [12]. There is also currently a lack of training in segmentation in the U.K. and international neurosurgical resident curricula [16].

To fill this gap, we offer a detailed practical guide for novices on how to segment common cerebral lesions. Specifically, we demonstrate a pipeline that makes use of ITK-SNAP to delineate meningiomas, subarachnoid haemorrhage and glioblastomas. We chose these three conditions as they represent distinct pathologies involving different segmentation methods. Hence, we demonstrate a wide variety of possible segmentation methods.

## 2. Materials and Methods

**Figure 1:**
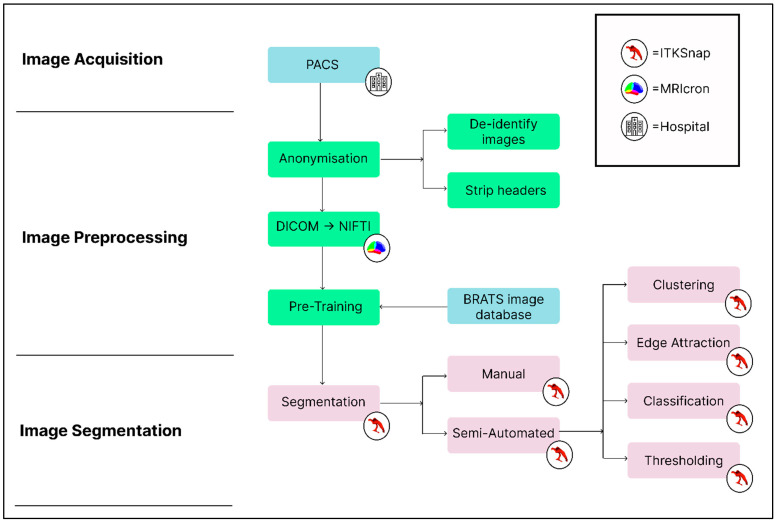
An example image-processing pipeline with image acquisition, pre-processing, segmentation and post-processing stages. (Single column with colour in print).

The image processing pipeline (Figure 1) summarises our process.

### 2.1. Ethics

This article was written as an educational guide and is exempt from ethical committee approval. Where relevant, patients approved the use of their scans for research and educational purposes.

### 2.2. Hardware

Segmentations were performed on an Hewlett-Packard Pavilion laptop 2015 (made in China) with an Advanced Micro Devices (AMD) 2.00 GHz processor and 16 GB of Random Access Memory (RAM). The time taken for image pre-processing averaged around 15–60 s. The manual segmentation of meningioma required 15 min, compared to 20 min and 30 min for GBM and SAH semi-automated segmentation, respectively.

### 2.3. Software

ITK-SNAP is an openly accessible and easy-to-use software that has powerful built-in semi-automated segmentation tools [17]. It is available with Windows, MacOS and Linux [18] and allows for image processing, segmentation and visualisation. 3D Slicer is a possible alternative software, which is a general-purpose 3D medical image analysis tool that can perform similar functions of contour-based segmentation [19].

MRIcron is a free cross-platform image viewer that can convert Digital Imaging and Communication in Medicine (DICOM) images to Neuroimaging Informatics Technology Initiative (NIfTI) format [20]. Matrix Laboratory (MATLAB) is a programming language and software that allows the calculation of post-segmentation error metrics, such as the Dice similarity coefficient, to evaluate the quality of segmentation [14,21].

### 2.4. Image Acquisition

Glioblastoma (GBM) scans were sourced from the Medical Image Computing and Computer Assisted Interventions Society (MICCAI) Multimodal Brain Tumour Segmentation Challenge (BRATS) [22,23,24,25,26,27]. This database provided the T1-weighted, T1-weighted + contrast, T2-weighted and T2 Fluid-attenuated inversion recovery (FLAIR) scans for each patient. It also provides a ground truth manual segmentation. The inclusion criteria involved a pathologically confirmed diagnosis of GBM and an available O6-methylguanine-DNA methyl transferase (MGMT) promoter methylation status. Demographically, 60% of the patients were male, while 40% were females [22,28].

The scans for SAH and meningiomas were obtained from consenting patients from our institution. These images were originally downloaded from the Picture Archival and Communication System (PACS, Figure 1), a hospital system that allows medical professionals to gain access to medical imaging [29]. All data used were anonymised. The SAH patient was a female in her early 70s at the time of the scan, while the patient with the meningioma was man in his early 40s, who had a tumour of grade II.

PACS images are typically stored in DICOM format: the standard for raw images obtained from medical scanners [30]. DICOM files are converted to the NIFTI format using software like MRIcron (Figure 1) [20,31].

### 2.5. Training

Before segmentation, segmenters often require training to recognise key radiological features of meningiomas, SAH and GBM, the key features of which are summarised in Table 1.

### 2.6. Segmentation

**Figure 2:**
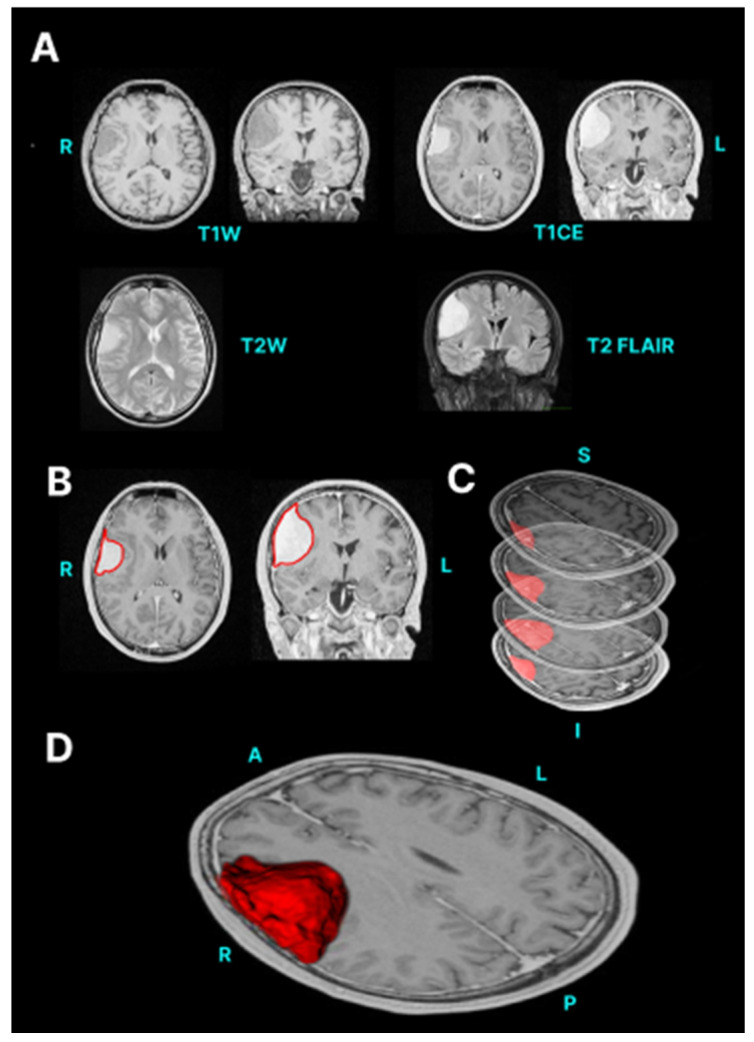
Manual segmentation of a convexity meningioma. (**A**) Original MRI. (**B**) Manual delineation of meningioma outline. (**C**) Interpolation of lesion through various slices. (single column with colour in print).

We utilise manual segmentations to delineate meningiomas, particularly given its complex morphology around the skull base. It involves annotating the full extent of the meningioma through a sequential array of MRI slices followed by interpolation to generate a 3D structure (Figure 2). A detailed step-by-step guide can be found in Supplementary Video S1 (introduction) and Supplementary Video S2 (meningioma segmentation).

**Figure 3:**
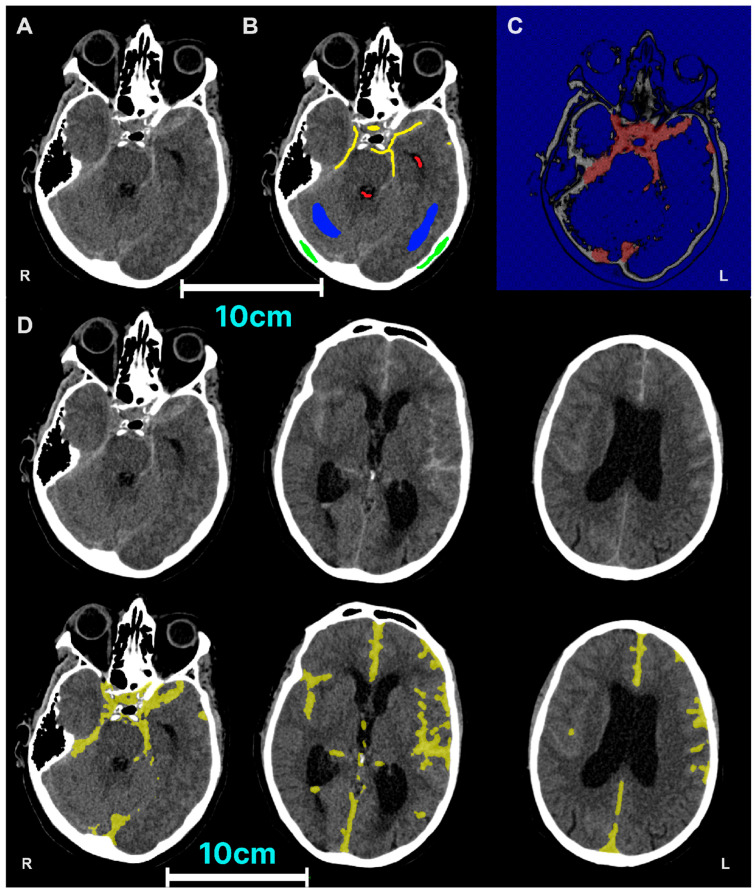
Semi-automated segmentation of SAH. (**A**) Original CT. (**B**) Manual labelling of different brain tissues, i.e., classification. Red represents cerebrospinal fluid, green represents bone, blue represents brain parenchyma and yellow represents subarachnoid haemorrhage. (**C**) Evolution of contours. (**D**) Final segmentation at different levels after manual inspection and editing. (Single column with colour in print).

We utilise semi-automated segmentation (classification) to delineate SAH (Figure 3A). In brief, different brain tissues are first manually labelled (Figure 3B). A contour is then initialised where the centre of the lesion is likely to be and grows stochastically to the lesion boundary (Figure 3C). Finally, the segmentation image generated is inspected and edited manually (Figure 3D, Supplementary Video S3). Particular attention is given to diffuse areas of SAH over the falx cerebri and adjacent to the venous sinuses.

**Figure 4:**
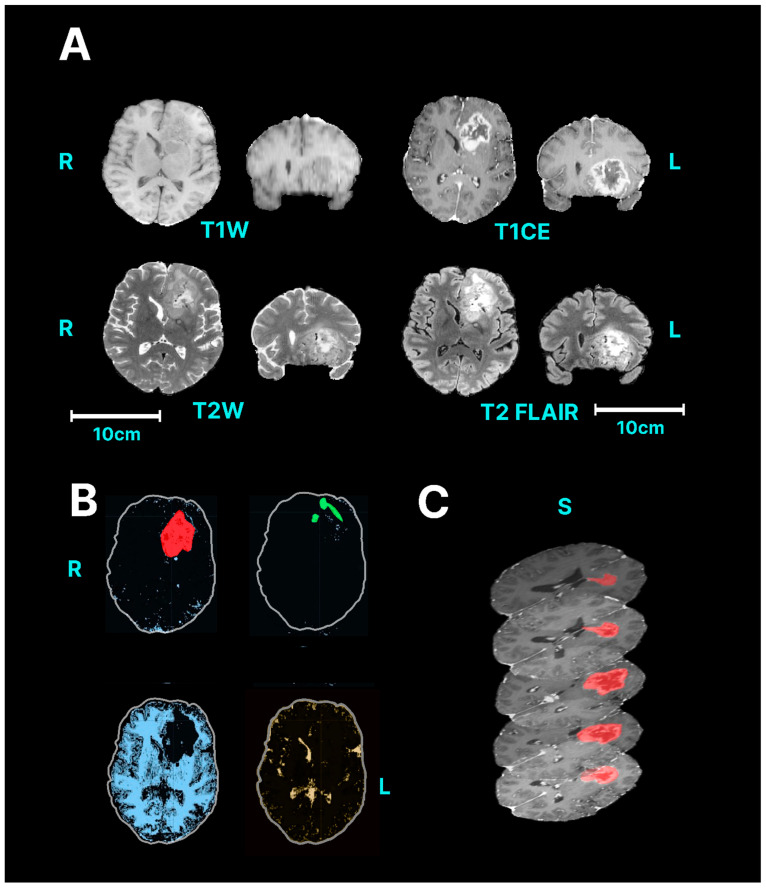
Semi-automated segmentation of a glioblastoma. (**A**) Original MRI. (**B**) Capturing the extent of GBM through classification. Red represents glioblastoma, green represents cerebral oedema, blue represents brain parenchyma and yellow represents cerebrospinal fluid. (**C**) Derivation of 3D lesion volume. (Single column with colour in print).

Using slight modifications of these steps, GBMs are segmented using a similar process (Figure 4, Supplementary Video S4). Particular attention is given to areas of oedema which may or may not be needed in the segmentation.

### 2.7. Methods of Quality Control

Quality control is integral to image segmentation in order to maintain the reliability, consistency and accuracy of derived radiomics [40]. In addition to segmenter training, commonly used quality control methods include the use of expert-defined segmentations, appropriate labelling and error metrics. 

#### 2.7.1. Expertly Defined Segmentations and the Imaging Ground Truth

During manual inspection, segmentations are generally checked by consultant radiologists, anatomists and other expert clinical neuroscientists to provide rigorous visual validation. This acts as a ground truth, which allows for a more reliable delineation of lesions and the exclusion of errors. If identified, the errors can then be fixed immediately with cross-checking [41]. However, there remains doubt as to what constitutes an ‘expert’ due to the varied knowledge base and techniques required [42].

#### 2.7.2. Error Metrics

The Dice similarity coefficient and the Jaccard index are two commonly used performance statistical metrics to evaluate the efficacy of segmentation [43], i.e., both can be applied to check for the consistency of segmentation for the same type of lesions against the ground truth or the inter-segmenter consistency. Both indices are calculated by comparing the segmentation performed by a particular method (A) against the gold standard (B, typically expert manual segmentation). These coefficients range between 0 and 1, with 1 indicating high similarity whereas 0 indicates complete separate results. 

While the Dice similarity coefficient more strongly weighs the commonalities between two objects and takes into account the total lesion volume, the Jaccard index penalises the differences between two objects and is not volume dependent.

### 2.8. Post-Segmentation Processing and Radiomics

After successful segmentation, quantitative features of the pathologies such as the size, shape, contrast-enhancement and texture (radiomics) can be extracted using pre-programmed algorithms [44]. A few examples of relevant radiomics features can be found in the following papers [45,46,47].

## 3. Results and Discussion

In this article, we provide a step-by-step guideline for the manual and semi-automated segmentation of three common neurosurgical pathologies: meningioma, subarachnoid haemorrhage and glioblastoma using ITK-SNAP.

Manual segmentation performed by expert surgeons and radiologists still currently remains the gold standard [48] and is particularly helpful when the lesion and surrounding tissue have similar signal intensities, causing automated algorithms to fail [49]. Semi-automated segmentation offers increased efficiency and improved repeatability while retaining an aspect of real-time quality control [9] and is particularly suitable for segmenting anatomically complex brain lesions where sparse training data sets exist [14].

Inexperienced trainees can quickly develop the ability to perform manual and semiautomated segmentation. In a particular study, a group of five participants, including a neurosurgeon, two biomedical engineers and two medical students, were given a standardised 10 min preparation time before segmenting four vestibular schwannoma scans using manual and semi-automated methods. Three of these participants were inexperienced in segmentation, while two were experts [9]. The inexperienced participants had a Dice score of 0.899 compared to 1.901 for the expert segmenters, against ground truth data. This suggests, albeit in a small sample, that segmentation skills can be trained over a short period of time [9].

Despite the advancements in semi-automated and automated methods, manual segmentation remains a commonly used method for segmentation and is typically held as a gold standard when performed by an expert [50]. Semi-automated segmentation, though faster and less labour-intensive, can miss out key areas of brain lesions due to heterogeneity between lesions. Thus, semi-automated methods may still require supervision by a clinician and extensive manual checks [8].

Automated segmentation methods exist for meningioma and have been relatively effective in demarcating lesion boundaries [51,52,53]. However, meningiomas often have areas of heterogeneity: oedema and necrosis make automatic classification methods difficult to implement [13]. Automated, quantitative tools for non-invasive sub-classification of meningiomas on multi-sequence MR images have recently become more available, following the publication of open access manually segmented datasets.

While automated segmentation further improves efficiency, they require extensive coding and training on large databases [13], and usually still require manual quality control steps. The lack of publicly available data for spatially complex brain lesions, such as subarachnoid haemorrhage, can limit their utility at present [54]. With greater capability for training, larger databases and more computational power, this will likely become a less pertinent issue [55].

While segmenting, special considerations should be paid to patient confidentiality. Article 9 of the EU general data protection regulation (GDPR), for example, prohibits the processing and revealing of data concerning health and biometric data for the purpose of uniquely identifying a natural person, unless the data subject has given explicit consent [56]. Medical image from PACS stored in the DICOM format contains patient’s protected health data in the header [57]. When converting DICOM files into NIFTI format using software like MRIcron [20], the data header should be anonymised. However, patients’ facial contour details still remain in the CT and MRI scans, and three-dimensional models of the patient’s facial appearance can be reconstructed [57]. This issue may be resolved by defacing/skull-stripping algorithms [58] or the application of digital masks [59].

If neurosurgery is to become more automated and personalised as anticipated [60], early delineation and characterisation of brain lesions are needed to triage and optimise management. This requires fast, accurate lesion segmentation which can be embedded into the clinical workflow. Such algorithms require training sets of segmentations at scale to ensure both accuracy and precision. By educating clinicians to confidently carry out manual and semi-automated segmentation, the underpinning training data will be accrued. Moreover, by performing segmentations, neurosurgical trainees can actively improve their understanding of the radiology of brain pathologies and mentally rehearse relevant operative procedures [12].

In conclusion, neurosurgical image segmentation has great potential within clinical care, education and research. Our educational guideline provides a step-by-step pathway for neurosurgical trainees new to medical image segmentation. This allows them to effectively apply this within their research and practice.

## Figures and Tables

**Table 1:** Key imaging sequences and radiological features of meningioma, glioblastoma and subarachnoid haemorrhage. CT: computed tomography, FLAIR: Fluid Attenuated Inversion Recovery. T1w: T1 weighted. T2w: T2 weighted. TIRM: Turbo Inversion Recovery Magnitude.

Lesion	Sequences for Segmentations	Radiological Features
Meningioma	T1w T1w + contrast T2w	Meningiomas have isointensity to slight hypointensity with T1 weighting. With T2-weighted sequences, meningiomas have isointensity to slight hyperintensity [32]. Two basic morphologies of meningioma include en plaque with a sheet-like dural extension and globose with a broad dural attachment [33]. The thick extended dura (commonly referred to as a dural tail) tends to extend away from the meningioma, which can be easily missed [34]. Bone changes may be visible, such as hyperostosis, osteolysis, enlargement of the skull base foramina and meningioma calcification [35].
Subarachnoid Haemorrhage (SAH)	CT non-contrast	Acute haemorrhage will be present with 15–25 Hounsfield Units (HU) of greater density than normal grey and white matter on a CT scan [36]. Anatomically, SAH is typically found present in the interpeduncular cistern, the Sylvian fissure, the occipital horns of the lateral ventricles and the deep sulci on each side of the medial longitudinal fissure [37].
Glioblastoma (GBM)	T1w T1w + contrast T2w T2 FLAIR/TIRM	GBMs are generally hyperintense on T2-weighted images but are hypo- or isointense on T1-weighted images [38]. GBM often have enhancing and non-enhancing components. Necrosis is typically visible as a low signal intensity (SI) on T1-enhanced MRI and located at the centre of the lesion [39]. Cystic components of a GBM are typically T2W hyperintense and T1 hypointense, with a well-defined thin wall. There can also an area of oedema surrounding the tumour that is visible in T2 FLAIR scans [38].

## Supplementary Materials

The following supporting information can be downloaded at: https://www.mdpi.com/article/10.3390/neurosci5030021/s1, Video S1: introduction. Video S2: meningioma segmentation. Video S3: SAH. Video S4: GBM.
